# Epistasis with HLA DR3 implicates the P2X7 receptor in the pathogenesis of primary Sjögren's syndrome

**DOI:** 10.1186/ar4248

**Published:** 2013-06-02

**Authors:** Susan Lester, Leanne Stokes, Kristen K Skarratt, Ben J Gu, Kathy L Sivils, Christopher J Lessard, James S Wiley, Maureen Rischmueller

**Affiliations:** 1 Department of Rheumatology, The Queen Elizabeth Hospital, Woodville South, South Australia, Australia; 2 Sydney Medical School Nepean, University of Sydney, Nepean Hospital, Penrith, NSW, Australia; 3 Health Innovations Research Institute, School of Medical Sciences, RMIT University, Bundoora, Victoria, Australia; 4 Florey Neuroscience Institutes, University of Melbourne, Parkville, Victoria, Australia; 5 Arthritis and Clinical Immunology Research Program, Oklahoma Medical Research Foundation, Oklahoma City, OK, USA; 6 Discipline of Medicine, University of Adelaide, South Australia, Australia

## Abstract

**Introduction:**

The aim of this study was to examine the association between functional polymorphisms in the pro-inflammatory P2X7 receptor and the Ro/La autoantibody response in primary Sjögren's syndrome (pSS).

**Methods:**

Twelve functional *P2RX7 *polymorphisms were genotyped in 114 pSS patients fulfilling the Revised American-European Consensus Criteria for pSS, and 136 controls. Genotyping of the A1405G (rs2230912) polymorphism was performed on a replication cohort consisting of 281 pSS patients and 534 controls. P2X7 receptor function in lymphocytes and monocytes was assessed by measurement of ATP-induced ethidium+ uptake. Serum IL-18 levels were determined by ELISA.

**Results:**

The minor allele of *P2RX7 *A1405G is a tag for a common haplotype associated with gain in receptor function, as assessed by ATP-induced ethidium+ uptake. A positive association between 1405G and anti-Ro±La seropositive pSS patients was observed in Cohort 1. Although not replicated in Cohort 2, there was a consistent, significant, negative epistatic interaction effect with HLA-DR3 in seropositive pSS patients from both cohorts, thereby implicating this gain of function variant in the pathogenesis of pSS. Serum IL-18 was elevated in seropositive pSS patients, but was not influenced by *P2RX7 *A1405G.

**Conclusions:**

The *P2RX7 *1405G gain-of-function haplotype may be a risk factor for seropositive pSS in a subset of subjects who do not carry HLA risk alleles, but has no effect in subjects who do (epistasis). Potential mechanisms relate to autoantigen exposure and inflammatory cytokine expression. The observed elevation of IL-18 levels is consistent with P2X7 receptor activation in seropositive pSS patients. Collectively these findings implicate P2X7 receptor function in the pathogenesis of pSS.

## Introduction

Primary Sjögren's syndrome (pSS) is a systemic autoimmune disease characterized by lymphocytic and monocytic infiltration into the salivary and lacrimal glands with inflammation, destruction of acini and failure of exocrine secretion. A wide range of extraglandular features also manifest in a subset of patients. Autoantibodies targeting the Ro and La components of a ribonucleoprotein (RNP) complex are highly specific for pSS and constitute one of the classification criteria for this disease [1]. Once considered an epiphenomenon, these autoantibodies are now recognized to be involved in the systemic inflammation of pSS [2].

A substantial polygenic component underlies susceptibility to the Ro/La autoantibody response in pSS [3,4], however the strongest individual association is observed with HLA-DRB1*03:01 (DR3, Chr 6p21.3) [5]. Additional genes are also likely to be involved, and one candidate is the P2X7 receptor (Chr 12q24.31), whose chromosomal location lies within mapped human and murine systemic lupus erythematosus (SLE) susceptibility loci [6]. The purinergic P2X7 receptor has been implicated in several murine models of autoimmune disease, including experimental autoimmune encephalomyelitis [7], rheumatoid arthritis (RA) [8] and autoimmune hepatitis [9].

The P2X7 receptor belongs to a two-transmembrane family of receptors, which are trimeric in the membrane and are attached via non-muscle myosin to the actin cytoskeleton [10-12]. Activation of P2X7, in response to the binding of extracellular ATP, opens a non-selective cation channel allowing an immediate influx of Ca2+ and Na+, and efflux of K+ ions. Continued activation, over tens of seconds, allows a larger permeability state to develop (termed pore formation), which is accompanied by extensive membrane blebbing, and ultimately cell death, with features of both apoptosis and necrosis [13,14]. Extracellular ATP is normally maintained at low nanomolar levels by a ubiquitous ecto-ATPDase (CD39), although in an inflammatory milieu, ATP may be released from dead or injured cells, as well as by cell swelling or from autonomic nerves [15].

The P2X7 receptor is found on many cells and tissues throughout the body. The receptor is most highly expressed on cells of the macrophage lineage, including dendritic cells and microglia [13,16], and both P2X7 receptors and HLA Class II molecules are upregulated in parallel when monocytes differentiate to these mature cell types. P2X7 receptors are also abundant on both acinar and ductal cells from submandibular and other salivary glands [17,18]. A special role for this receptor in salivary glands has been highlighted by its direct involvement in the regulation of fluid secretion [19,20]. In cells of the monocyte-macrophage lineage, there is much evidence that P2X7 is pro-inflammatory [16,21], and P2X7 gene-deleted mice show major reductions in cytokine responses to various inflammatory stimuli [8]. Activation of the P2X7 receptor results in maturation and release of the pro-inflammatory cytokines, IL-1β and IL-18 [21-23], a response that has been linked to formation of the cryopyrin (NALP3) inflammasome and subsequent caspase-1 activation [21,24]. Elevated serum levels of both IL-1β and IL-18 have been reported in pSS [25,26], and these cytokines have been implicated in salivary gland pathology in both mouse models [27] and human disease [28,29].

There is considerable variability in P2X7 function between individuals, which can be largely attributed to polymorphisms of the *P2RX7 *gene. To date, one dozen polymorphisms have been identified in *P2RX7*, which alter receptor function [30,31]. We and others have shown that polymorphisms that increase or decrease P2X7 receptor function confer a corresponding increase or decrease in ATP-stimulated secretion of IL-1β or IL-18 from monocytes primed with lipopolysaccharide [31-33]. We have now examined these functional *P2RX7 *SNPs in patients with pSS, and have identified an epistatic interaction between the major *P2RX7 *gain-of-function haplotype and HLA-DR3 in patients with seropositive pSS.

## Materials and methods

### Study participants

Cohort 1 included 114 Caucasian pSS patients (88% female) from the South Australian Sjögren's Syndrome research registry, and 136 Caucasian population-based controls. The replication cohort (cohort 2) included 281 Caucasian pSS patients (95% female) obtained from the Oklahoma Medical Research Foundation (OMRF) plus 534 controls.

All patients met the revised 2002 American-European consensus research classification criteria for pSS [1]. Anti-Ro±La status was performed as part of routine diagnosis, and was available for all patients. Autoantibody testing using recombinant protein was performed by ELISA in cohort 1 [4], and by immunodiffusion in cohort 2 [34]. The majority of patients were autoantibody-positive, with 82% of pSS patients from the first cohort, and 71% of pSS patients from the second cohort positive for anti-Ro±La.

HLA DR3 status was available for all patients and controls for cohort 1 (medium resolution *DRB1 *genotyping performed by the Tissue Typing Laboratory, Adelaide Red Cross Blood Transfusion Service, South Australia), and for the majority of pSS patients from cohort 2 (a DR3 sequence-specific priming assay). A DR3 proxy SNP, rs2187668 [35], was used for analyses of the controls from cohort 2. The use of the minor allele of rs2187668 as a proxy for DR3 was validated against individuals with known DR3 status in each cohort, with observed kappa agreements of 0.95, and 0.92 respectively (data not shown).

The study was approved by the Human Ethics Committees of The Queen Elizabeth Hospital, the Sydney West Area Health Service and OMRF, in accordance with the Helsinki Declaration, and all participants gave informed, written consent.

### *P2RX7 *SNP genotyping

Twelve SNPs (one intron splice site, and eleven non-synonymous) throughout the *P2RX7 *gene, most of which have been previously characterised as exerting either loss- or gain-of- function over the wild-type receptor, were examined in pSS patients and controls from cohort 1 (Table 1). Eight SNPs were analysed by high-throughput TaqManTM genotyping assays performed on an ABI Prism ABI Prism^®^7900HT sequence detection system (Applied Biosystems, Foster City, CA, USA) at the SUPAMAC Facility, Royal Prince Alfred Hospital, Sydney, Australia. G1068A was genotyped using the commercial TaqManTM assay, C_11704039_10 (Applied Biosystems). SNPs G151T, G474A, G946A, C1096G, A1405G, A1513C and T1729A were genotyped using primers and probes as listed in Additional file 1, Table S1. Four SNPs, T253C, C489T, G835A and G853A were genotyped by a homogeneous mass extension assay at the Australian Genome Research Facility (St Lucia, Queensland).

**Table 1:** *P2RX7 *SNPs genotyped in pSS patients and normal subjects

#	ID	SNP	Exon	Protein	**Functional effect of minor allele **[30,31,40]
1	rs35933842	G151T	1	Intron 1 splice site	Loss
2	rs17525809	T253C	2	V76A	Partial loss
3	rs28360447	G474A	5	G150R	Loss
4	rs208294	C489T	5	H155Y	Gain
5	rs7958311	G835A	8	R270H	Partial loss
6	rs7958316	G853A	8	R276H	Loss
7	rs28360457	G946A	9	R307Q	Loss
8	rs1718119	G1068A	11	A348T	Gain
9	rs2230911	C1096G	11	T357S	Partial loss
10	rs2230912	A1405G	13	Q460R	Gain^a^
11	rs3751143	A1513C	13	E496A	Loss
12	rs1653624	T1729A	13	I568N	Loss

^a^The minor allele 1405G is a tag for haplotype 4 (Figure 1). pSS, primary Sjögren's syndrome; SNP, single nucleotide polymorphism.

Genotype data for the P2X7 A1405G SNP (rs2230912) and the DR3 proxy, rs2187668 were obtained for cohort 2 using the Illumina OMNI-1 Quad array (San Diego, CA, USA) following manufacturer's protocols. These subjects were also evaluated for possible population stratification using principal component analysis implemented in Eiganstrat [36] utilizing approximately 30,000 independent SNPs (r2 <0.20) scattered thought the genome. Samples were plotted alongside the HapMap populations to determine which ones were genetic outliers.

### Serum IL-18 assays

Serum IL-18 levels were assayed by sandwich ELISA (Human IL-18 ELISA Kit, Medical and Biological Laboratories Company Limited, Nagaoya, Japan, catalogue number 7620), according to the manufacturer's instructions.

### P2X7 function measured by ATP-induced ethidium uptake (pore assay)

Peripheral venous blood was obtained from healthy volunteers and mononuclear cells were isolated by Ficoll-Paque gradient centrifugation. P2X7 receptor function was measured by the ATP-induced ethidium+ uptake in a mixed cell (B and T lymphocytes and monocytes) suspension using time-resolved two-colour flow cytometry [37]. Briefly, mononuclear cells were labelled with a phycoerythrin (PE)-conjugated monoclonal antibody to CD3 and an allophycocyanin (APC)-conjugated monoclonal antibody to CD14 (Dako). Ethidium+ (25 μM) was added to a cell suspension maintained at 37°C with stirring in a time-zero module, and 1 mM ATP was added 40 s later. The cells were analysed at 1000 events/s on a FACSCalibur flow cytometer and gated by forward and side scatter and by cell type-specific monoclonal antibody. The linear mean channel of fluorescence intensity for each gated subpopulation over successive 10-s intervals was analysed by WinMDI version 2.8 software [38] and plotted against time to estimate the maximum rate of ethidium+ uptake. The linear portion of the ethidium+ uptake plot was used for slope calculations over a period of 1 minute.

### Statistical analysis

Differences in allele frequencies between patients and controls were analysed by logistic regression using the default additive model for allele coding and odds ratios (OR) were derived from exponentiation of the regression coefficients. Epistasis was analysed using a two-factor interaction logistic regression model, with the significance of the epistasis determined by the significance of the coefficient for the interaction term. Analyses for cohort 2 were ancestry-adjusted by the inclusion of the principal components as covariates. Serum IL-18 levels were log-normally distributed and were therefore analysed by lognormal regression and data presented as medians. These analyses were performed using Statistica v6.1 (StatSoft Inc, Tulsa, Oklahoma, USA). Haplotype inference was performed using Phase version 2 software [39].

## Results

### Functional *P2RX7 *polymorphism in pSS patients and population based controls

The minor allele frequencies for each of the 12 *P2RX7 *functional SNPs in pSS patients and controls from cohort 1 are presented in Additional file 1, Table S2. The minor alleles of five SNPs (G151T, G474A, G853A, G946A and T1729A) were rare (frequency <5%). There was no evidence of an association between any of the 12 functional SNPs and pSS. However, the frequency of the minor allele of the A1405G SNP (rs2230912) was quite discrepant between seronegative and seropositive pSS patients in cohort 1, and was in fact increased in seropositive pSS patients compared to controls (OR = 1.78, 95% CI 1.09, 2.92, *P *= 0.02) (Table 2). However this association was not confirmed in cohort 2.

**Table 2:** Frequency of the minor allele of *P2RX7 *A1405 G allele in pSS patients and controls

Cohort	Group	Number	A1405G MAF^a^	Odds ratio (95% CI)	*P*-value
Cohort 1	Controls	136	0.15	1	
	All pSS	114	0.20	1.46 (0.91, 2.34)	0.12
	Seronegative pSS	19	0.07	0.44 (0.13,1.52)	0.22
	Seropositive pSS	95	0.23	1.78 (1.09, 2.92)	0.02
Cohort 2	Controls	534	0.15	1	
	All pSS	281	0.17	1.07 (0.79, 1.45)^b^	0.64
	Seronegative pSS	82	0.21	1.35 (0.87, 2.10) ^b^	0.18
	Seropositive pSS	199	0.16	0.96 (0.68, 1.36) ^b^	0.83

^a^MAF, minor allele frequency; ^b^principal components adjusted analysis; pSS, primary Sjögren's syndrome.

### The minor allele of *P2RX7 *A1405G exhibits a negative epistatic interaction with HLA DR3 in Ro±La seropositive pSS patients

We further investigated the role of the A1405G allele in anti-Ro±La seropositive pSS by considering epistasis with HLA DR3. The HLA region confers a strong genetic risk for seropositive pSS, and *DRB1 *alleles are markers of this risk. In Caucasian populations, the strongest association is observed with HLA DR3, although other *DRB1 *alleles are also involved, and the associated alleles are known to differ across different racial groups.

The joint distribution of HLA DR3 and the *P2RX7 *1405G allele was examined by a two-factor interaction logistic regression model in both cohorts. Differences in the available data in relation to ascertainment of DR3 status necessitated several analyses, and the results of the regression coefficients for these analyses are depicted in Table 3.

**Table 3:** Epistasis between *P2RX7 *1405G and HLA DR3 in anti Ro±La seropositive primary Sjögren's syndrome (pSS)

	Seropositive pSS (*n *= 95) versus controls (*n *= 136)			Seropositive pSS (*n *= 199) versus controls (*n *= 534)			Seropositive (*n *= 191) versus seronegative pSS (*n *= 80)			Seronegative pSS (*n *= 82) versus controls (*n *= 534)		
	Cohort 1: *DRB1*-typing			Cohort 2: DR3 proxy SNP^a^			Cohort 2: DR3 SSP^a^			Cohort 2: DR3 proxy SNP^a^		
Term	β	SE	*P*	β	SE	*P*	β	SE	*P*	β	SE	*P*
β0 (Intercept 1)	-1.64	0.28	<0.001	-0.01	2.64	1	-3.97	4.31	0.36	1.04	3.32	0.75
β1 (DR3-pos)	2.23	0.37	<0.001	1.35	0.21	<0.001	1.39	0.37	<0.001	0.28	0.33	0.40
β2 (*P2RX7*_1405G)	1.11	0.38	0.003	0.26	0.22	0.24	0.25	0.34	0.46	0.29	0.27	0.28
β3 (DR3*1405G)	-1.27	0.53	0.016	-0.76	0.35	0.027	-1.27	0.56	0.023	0.02	0.47	0.96

^a^Analysis performed using principle components adjustment for possible ancestry differences. The combined effect of these alleles was examined by two-factor interaction logistic regression (DR3*1405G) in both cohorts for the data tabulated in Additional File 1, Table S3. DR3 was coded positive (pos) or negative, and additive genetic coding was used for 1405G. The regression coefficients β1 and β2 represent the log^e ^odds ratios for individuals who carry DR3 alone and 1405G alone, relative to individuals who carry neither. The interaction term, β3, represents the deviation from the expected log odds ratio for individuals who carry both alleles, and is the test for epistasis between 1405G and DR3. Different analyses were required to accommodate differences in the available data for DR3 status. The results demonstrate that DR3 (β2), or proxy, is a significant risk factor for seropositive pSS, but not seronegative pSS. Further, there is a negative epistatic interaction between DR3 (or proxy) and *P2RX7 *1405G (β3) specifically in seropositive pSS. However, the association of the 1405G allele with seropositive pSS (in the absence of DR3, β2), observed in Cohort 1, was not replicated in Cohort 2. SE, standard error.

These analyses confirm that HLA DR3 is a strong risk factor for seropositive, but not seronegative pSS. However this association with DR3 appears somewhat greater in cohort 1 compared to cohort 2, which used a proxy SNP for DR3 status. For example, the regression coefficients (log-OR) reported in Table 3 equate to OR of 9.3 (95% CI 4.5, 19.2) for cohort 1, compared to 2.6 (95% CI 2.6, 5.8) for cohort 2. Further, there is remarkably consistent evidence of a statistically significant negative epistatic interaction between HLA DR3 (or proxy) and the *P2RX7 *1405G allele in seropositive pSS patients, whether comparing these patients to controls (in both cohorts), or seropositive pSS to seronegative pSS patients (sufficient data for cohort 2 only) (Table 3). This negative epistasis, which is assessed by β3 coefficient in Table 3, implies that the proportion of seropositive pSS patients who carry both alleles is significantly less than expected. However, the association between the *P2RX7 *1405G allele and seropositive pSS patients who do not carry DR3 (the β2 coefficient in Table 3) observed in cohort 1 (OR 3.0, 95% CI 1.4, 6.4) was not replicated in cohort 2 (OR 1.3, 95% CI 0.8, 2.0). This epistasis effect with DR3 was specific for the *P2RX7 *1405G allele, and was not observed with any other *P2RX7 *SNPs genotyped in cohort 1 (data not shown).

The most plausible interpretation of these findings is that the *P2RX7 *1405G allele is a risk factor for seropositive pSS in a specific subgroup of patients who do not carry HLA risk alleles, but confers no additional risk in individuals who do. In our analysis, the use of HLA DR3 would only partially adjust for the HLA risk, as other *DRB1 *alleles are also involved [3]. Given that the OR for the association with DR3 are greater in cohort 1 than in cohort 2 (Table 3), this implies that other *DRB1 *alleles may be relatively more important in cohort 2, yet the analysis could not adjust for this. This difference in HLA risk adjustment between cohorts may be sufficient to explain the lack of replication of the *P2RX7 *1405G allele association in cohort 2 (Table 3).

### The 1405G allele is a tag for a common *P2RX7 *haplotype

**Figure 1:**
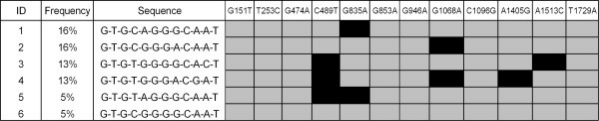
**Common (>5% frequency) *P2RX7 *haplotypes identified in the study population**. Grey squares represent the major alleles and black squares the minor alleles, for each single nucleotide polymorphism. Numbering of polymorphisms are based on the original mRNA sequence [Y09561.1; GenBank]. Six major haplotypes were identified with a combined frequency of 70%.

There was evidence of strong linkage disequilibrium between the *P2RX7 *SNPs. Haplotype reconstruction of pSS patients in cohort 1 and controls identified six major haplotypes (individual frequencies greater than 5%), with a combined frequency of 70% (Figure 1). These haplotypes and frequencies are comparable to those identified in a large Australian Caucasian cohort [30,31]. Importantly, the 1405G minor allele was observed on only one major haplotype (haplotype 4, frequency 13%), and this minor allele is therefore a tag for haplotype-4 which also carries minor alleles for both C489T and G1068A.

### The 1405G allele is associated with gain-of-function of the P2X7 receptor

**Figure 2:**
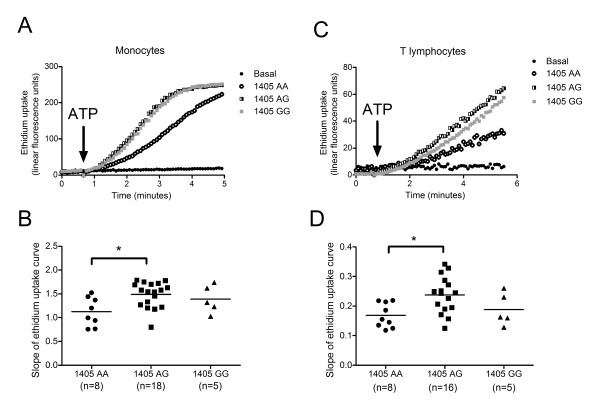
**Human peripheral blood mononuclear cells were analysed for ATP-induced ethidium uptake using time-resolved flow cytometry**. Monocytes were identified by an anti-CD14 APC antibody and T lymphocytes were identified by an anti-CD3-PE antibody. Ethidium uptake (25 μM) was measured in response to P2X7 receptor stimulation by 1mM ATP. (**A**) and (**C**) are representative ethidium uptake curves from genotyped individuals. (**B**) and (**D**) are scattergrams of collated data from 5 to 18 subjects from each genotype group (number of subjects shown in brackets) with ATP-induced ethidium uptake calculated from the slope of the linear portion of the uptake plot over 1 minute. *P < 0.05 from one-way analysis of variance with Dunnett's post hoc test.

P2X7 receptor function associated with the A1405G polymorphism was studied in monocytes and lymphocytes from peripheral blood of healthy individuals by measuring membrane pore formation (assessed by ethidium uptake), following receptor activation by ATP. Monocytes from individuals homozygous or heterozygous for the 1405G allele exhibited an increase in ATP-induced ethidium uptake compared with monocytes from homozygous 1405AA individuals (*P *= 0.018, *n *= 5 to 18 individuals) (Figure 2A, C), indicative of a gain in receptor function associated with the 1405G allele. Similar results were obtained with T-lymphocytes, where cells from 1405AG heterozygous individuals, exhibited an increased ATP-induced ethidium uptake (*P *= 0.015, *n *= 5 to 16 subjects) (Figure 2B, D). These results confirm previous reports that the 1405G allele is associated with gain of P2X7 receptor function [40].

### Serum IL-18 levels are increased in pSS patients with Ro/La autoantibodies

**Figure 3:**
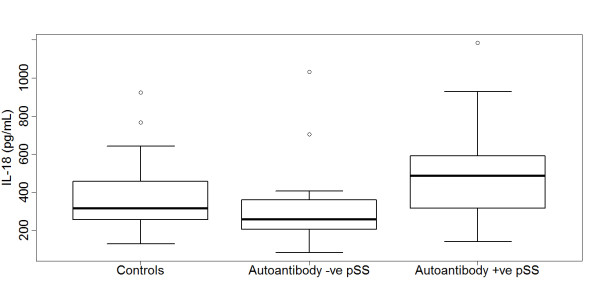
**Boxplots of serum IL-18 levels (pg/mL) in controls (*n *= 36), autoantibody negative pSS (*n *= 18), and autoantibody positive primary Sjögren's syndrome (pSS) (*n *= 54)**. Serum IL-18 levels were significantly different between the three groups (*P *= 0.0003, Kruskal-Wallis test), and highest in autoantibody-positive pSS.

IL-18 is a downstream cytokine released following P2X7 receptor activation, and serum IL-18 levels were measured in 72 pSS patients (54 seropositive for anti-Ro±La autoantibodies) and 36 age- and gender-matched controls from cohort 1 (Figure 3). When analysed by log-normal regression, mean serum IL-18 levels (in pg/mL) were 339, 318, and 498 for the controls, seronegative pSS and seropositive pSS patients, respectively. Serum IL-18 levels were almost 50% higher in Ro/La autoantibody-positive pSS patients compared to normal subjects (ratio = 1.47, 95% CI 1.22, 4.26, *P *< 0.001), consistent with previous reports. In contrast, the serum IL-18 levels in seronegative pSS patients were not different to that observed in normal controls (ratio = 1.12, 95% CI = 0.87, 1.45, *P *= 0.4). Among the seropositive pSS patients, there was no difference between the median IL-18 level in patients who carried the 1405G allele (549 pg/mL, *n *= 25) compared to patients with 1405A (460 pg/mL, *n *= 29, *P *= 0.13). There was no evidence of any influence of HLA DR3 status on IL-18 levels in these patients.

## Discussion

Autoantibodies, which target intracellular antigens, are a feature of systemic autoimmune diseases such as pSS. These autoantibodies react with autoantigen exposed on apoptotic or necrotic cells, or their debris, and resulting immune complexes promote chronic systemic inflammation via upregulation of the type I interferon system [2]. Delayed or defective clearance of apoptotic/necrotic cellular debris is understood to be an important factor in the breakdown of self-tolerance and ongoing immune complex mediated inflammation [41]. Ro±La autoantibodies are typically present in the majority of pSS patients (70 to 82% in the present study) and are associated with higher levels of glandular inflammation, and extraglandular features such as vasculitis, interstitial lung disease and lymphoma [42]. There is increasing evidence that autoantibody-positive pSS has different genetic risk factors to autoantibody-negative pSS [3,4], which possibly reflects different mechanisms of disease pathogenesis.

In this study we examined the interaction of the pro-inflammatory P2X7 receptor in the pathogenesis of Ro±La autoantibody-positive pSS (seropositive pSS) in two cohorts. The primary focus was on the *P2RX7 *1405G allele, which is a tag for a common haplotype conferring gain-of-function on the P2X7 receptor [30,31]. This haplotype also carries minor alleles from two other SNPS, 489T and 1068A (Table 1, Figure 1), and analysis of the functional effects of each in isolation suggests that they contribute, in an additive way, to the increased receptor function observed with this haplotype [30,43]. We have demonstrated an interaction between this gain of function SNP/haplotype and seropositive pSS, whereas no associations were observed with loss of function SNPs/haplotypes. Previous studies of *P2RX7 *polymorphism and systemic autoimmune disease have reported no association between the A1513C loss-of-function SNP in Caucasian patients with SLE or RA [44,45], which is consistent with the results of the present study.

We observed a remarkably consistent negative epistatic interaction between *P2RX7 *1405G and HLA DR3, the primary genetic risk factor for pSS, in two cohorts of seropositive pSS patients. This negative epistatic interaction was present when seropositive pSS patients were either compared to controls, or to seronegative pSS patients, so that the combined risk for individuals who carry both genetic factors is somewhat less than expected. In cohort 1 we also observed that the *P2RX7 *1405G allele is a risk factor for seropositive pSS in individuals who do not carry HLA DR3, but this effect was not replicated in cohort 2. The analysis in cohort 2 included principal components (derived from unrelated genetic markers) as covariates to adjust for possible population stratification, which can cause spurious associations in disease studies [36]. Therefore, the observed epistasis cannot be attributed to systematic ancestry differences between cases and controls.

The most plausible interpretation of these findings is that the *P2RX7 *1405G allele is a risk factor for seropositive pSS in a specific subgroup of patients who do not carry HLA risk alleles, but confers no additional risk in individuals who do, and that differences in results between the cohorts may reflect differences in adjustment for the HLA associated risk, which is only partially reflected by DR3 status. For example, it is unclear whether the use of a surrogate SNP for determining HLA-DR3 status in the cohort 2 controls may have influenced the results by underestimating the prevalence of HLA-DR3; further, HLA-DR2 is also associated with autoantibody-positive pSS [3], but this information was not available for cohort 2.

The epistatic interaction between DR3 and the *P2RX7 *1405G allele in seropositive pSS implies overlap in pathogenic mechanisms associated with these alleles. Unfortunately, the pathogenic mechanisms underlying HLA-associated disease susceptibility are not understood, therefore, the interpretation is highly speculative. We propose that the observed epistasis between DR3 and the *P2RX7 *1405G allele may reflect increased autoantigen exposure as a susceptibility mechanism for seropositive pSS.

Complement deficiency is implicated in the defective clearance of apoptotic/necrotic debris and, therefore, increased autoantigen exposure, in systemic autoimmune disease [46]. It has been proposed that this mechanism may contribute to some of the pSS disease susceptibility associated with HLA DR3, which is in linkage disequilibrium with a C4 null allele [47]. P2X7 receptor activation may also contribute to increased autoantigen exposure in systemic autoimmunity. In the presence of the ATP ligand, P2X7 receptor activation may result in the induction of cell membrane blebbing and release of microparticles. Prolonged activation of the P2X7 receptor by ATP results in cell death with both apoptotic/necrotic features [13,14], which has also been demonstrated in rat parotid acinar ParC5 cells [48], accompanied by autoantigen cleavage [49]. Importantly, the A1513C loss of function SNP is associated with a decrease in ATP-induced apoptosis in CD4(+) lymphocytes isolated from SLE patients [45], although the gain-of-function polymorphism has not yet been evaluated. Membrane microparticles released by the P2X7-induced blebbing process contain not only P2X7 receptors but also Ro-52, a component of the P2X7 membrane complex both in THP-1 macrophages and transfected HEK-293 epithelial cells [10], and also a target of autoantibodies in pSS. Further, we have recently demonstrated that P2X7, in the absence of ATP, acts as a scavenging receptor for apoptotic cellular debris [50], although the downstream effects of this have not been elucidated.

In addition to the genetic epistasis, our data suggest that there is P2X7 receptor activation in seropositive pSS. Activation of P2X7 is known to lead to processing and secretion of pro-inflammatory cytokines IL-1β and IL-18 from monocyte/macrophages via activation of the NALP3 inflammasome, which is thought to play a role in a spectrum of inflammatory diseases [51], including rheumatic inflammatory diseases [52]. We have demonstrated elevated serum IL-18 levels in Ro±La autoantibody-seropositive pSS patients, and activation of the NALP3 inflammasome, via P2X7, may be one of the mechanisms involved. There was no evidence for an effect of DR3 or 1405G status on IL-18 levels, although patient numbers were limited. Previous studies in systemic inflammatory diseases have demonstrated that the ATP-induced monocyte P2X7 receptor response (as measured by IL-1β secretion) is attenuated in SLE, but enhanced in RA [45,53], and that this response is influenced by *P2RX7 *polymorphism. However, the A1405G SNP has not been evaluated in this context.

Aside from its pro-inflammatory effects, ATP activation of the P2X7 receptor has also emerged as an important regulator of autonomic function, including salivary gland secretion. While P2X7 receptor activation can stimulate secretion in mouse submandibular bands, activation also results in the inhibition of muscarinic receptor-induced fluid secretion [19]. Therefore, activation of P2X7 in the milieu of salivary gland inflammation in pSS may contribute to the failure of fluid secretion irrespective of the extent of glandular destruction, through interference of autonomic signalling, a well-documented phenomenon in pSS. This dysregulation of autonomic function may also be implicated in the clustering of autonomic symptoms we have previously reported in pSS [54], and further research into the role of P2X7 and autonomic dysfunction in pSS is warranted.

## Conclusions

In summary, we have identified that the *P2RX7 *1405G gain-of-function allele exhibits a negative epistatic interaction with HLA DR3 on the risk of autoantibody-positive pSS. This, coupled with other studies, implicates P2X7 receptor function in the pathogenesis of primary Sjogren's syndrome.

## Abbreviations

APC: allophycocyanin; ELISA: enzyme-linked immunosorbent assay; IL: interleukin; NALP3: cryopyrin; OMRF: Oklahoma Medical Research Foundation; OR: odds ratio; PE: phycoerythrin; pSS: primary Sjögren's syndrome; RA: rheumatoid arthritis; RNP: ribonucleoprotein; SLE: systemic lupus erythematosus; SNP: single nucleotide polymorphism.

## Competing interests

The authors declare that they have no competing interests.

## Authors' contributions

SL: study design, genotyping, data analysis, interpretation, manuscript preparation; LS: ethidium uptake assays, analysis and manuscript preparation; KS: genotyping, IL-18 assays, manuscript preparation; BG: study design, manuscript preparation; KS: genotyping, interpretation and manuscript preparation; CL: genotyping, interpretation and manuscript preparation; JW: study concept and design, interpretation and manuscript preparation; MR: study concept and design, interpretation and manuscript preparation. All authors have read and approved the final manuscript.

## Supplementary Material

Additional file 1 **Table S1 showing custom Taqman genotyping primers and probes. Table S2 showing *P2RX7 *minor allele frequency in pSS patients (cohort 1, *n *= 114) and normal subjects (*n *= 136)**. Table S3 showing Genotyping results for *P2RX7 *A1405G for seropositive pSS, seronegative pSS and control subjects in cohort 1 and cohort 2. Click here for file
