# Supplementary material for: Epistasis with HLA DR3 implicates the P2X7 receptor in the pathogenesis of primary Sjögren's syndrome
Source: Arthritis Res Ther. 2013 Jun 2;15(4):R71. doi: 10.1186/ar4248 (PMC3979150; doi:10.1186/ar4248)
Supplement: Additional file 1 — Table S1 showing custom Taqman genotyping primers and probes. Table S2 showing P2RX7 minor allele frequency in pSS patients (cohort 1, n = 114) and normal subjects (n = 136). Table S3 showing Genotyping results for P2RX7 A1405G for seropositive pSS, seronegative pSS and control subjects in cohort 1 and cohort 2. [file ar4248-S1.DOCX]

Table S1. Custom Taqman genotyping primers and probes

| SNP ID | Forward Primer | Reverse Primer | VIC labelled Probe | FAM labelled Probe |
| --- | --- | --- | --- | --- |
| rs35933842 (G151T) | 5′-ttcttccacgtgatcatcttttcct-3′ | 5′-cggccactgcagagatctg-3′ | 5′-cccacttaccaaacgt-3′ | 5′-cccacttaacaaacgt-3′ |
| rs28360447 (G474A) | 5′-caggtcttctggttcccttcat-3′ | 5′-cgatgctttgacccctataggaat -3′ | 5′-tcagaccggaaggtg-3′ | 5′-cagaccagaaggtg-3′ |
| rs28360457 (G946A) | 5′-cagatacgccaagtactacaaggaa-3′ | 5′-tcaaaacggatcccgaagacttt-3′ | 5′-atgttgagaaacggactc-3′ | 5′-atgttgagaaacagactc-3′ |
| rs2230911 (C1096G) | 5′- gggagcgacagcagttactg-3′ | 5′-cagcgcttgtctgcattctc-3′ | 5′-ctcatcgacacttactc-3′ | 5′-tcatcgacagttactc-3 |
| rs2230912 (A1405G) | 5′-cccgattcctggacaacca-3′ | 5′-gctatccctggatctaggagtcg-3′ | 5′-tttctaagcagctgtatct-3′ | 5′-ctaagcagccgtatct-3′ |
| rs3751143 (A1513C) | 5′-gctgcctcccatctcaactc-3′ | 5′-gctctgaggtggtgatgca-3′ | 5′-agcacagctcctccag-3′ | 5′-cacagcgcctccag-3′ |
| rs1653624 (T1729A) | 5′-cttcggctcccaggacatg-3′ | 5′-cctccagcggcagca-3′ | 5′-ctttgccatcctgcc-3′ | 5′-tttgccaacctgcc-3′ |

Table S2: *P2RX7* Minor Allele frequency in pSS patients (cohort 1, N = 114) and normal subjects (N = 136)

| # | SNP | Minor Allele | pSS | Controls | Exact p-value |
| --- | --- | --- | --- | --- | --- |
| 1 | G151T | T | 1% | 1% | 0.69 |
| 2 | T253C | C | 5% | 6% | 0.69 |
| 3 | G474A | A | 0% | 1% | 0.39 |
| 4 | C489T | T | 46% | 40% | 0.18 |
| 5 | G835A | A | 26% | 28% | 0.55 |
| 6 | G853A | A | 3% | 5% | 0.35 |
| 7 | G946A | A | 1% | 1% | 0.71 |
| 8 | G1068A | A | 39% | 39% | 0.92 |
| 9 | C1096G | G | 7% | 6% | 0.86 |
| 10 | A1405G | G | 19% | 15% | 0.24 |
| 11 | A1513C | C | 20% | 19% | 0.82 |
| 12 | T1729A | A | 2% | 2% | 0.76 |

Table S3: Genotyping results for P2RX7 A1405G for seropositive pSS, seronegative pSS and control subjects in cohort 1 and cohort 2.

Cohort 1: Seropositive pSS vs Controls:

| GROUP | DR3 | P2X7 A1405G | | | TOTAL |
| --- | --- | --- | --- | --- | --- |
|  |  | AA | AG | GG |  |
| Seropositive pSS | Pos | 45 | 18 | 3 | 66 |
| Seropositive pSS | Neg | 14 | 13 | 2 | 29 |
|  | Total | 59 | 31 | 5 | 95 |
| Control | Pos | 23 | 16 | 0 | 39 |
| Control | Neg | 76 | 19 | 2 | 97 |
|  | Total | 99 | 35 | 2 | 136 |

Cohort 2: Seropositive pSS vs Seronegative pSS (DR3 by SSP):

| GROUP | HLADR3 | P2X7 A 1405G | | | Total |
| --- | --- | --- | --- | --- | --- |
|  |  | AA | AG | GG |  |
| Seropositive pSS | Positive | 76 | 26 | 0 | 102 |
| Seropositive pSS | Neg | 55 | 32 | 2 | 89 |
|  | Total | 131 | 58 | 2 | 191 |
| Seronegative pSS | Positive | 14 | 11 | 1 | 26 |
| Seronegative pSS | Neg | 36 | 16 | 2 | 54 |
|  | Total | 50 | 27 | 3 | 80 |

Cohort 2: Seropositive pSS, Seronegative pSS vs Controls (DR3 proxy SNP^1^):

| Group | rs2187668 (DR3 proxy) | P2X7 A1405G | | | Total |
| --- | --- | --- | --- | --- | --- |
|  |  | AA | AG | GG |  |
| Seropositive pSS | GG | 62 | 32 | 2 | 96 |
| Seropositive pSS | AG | 72 | 28 | 0 | 100 |
| Seropositive pSS | AA | 3 | 0 | 0 | 3 |
|  | Total | 137 | 60 | 2 | 199 |
| Seronegative pSS | GG | 37 | 19 | 2 | 58 |
| Seronegative pSS | AG | 13 | 8 | 1 | 22 |
| Seronegative pSS | AA | 1 | 1 | 0 | 2 |
|  | Total | 51 | 28 | 3 | 82 |
| Control | GG | 293 | 105 | 9 | 407 |
| Control | AG | 84 | 32 | 3 | 119 |
| Control | AA | 4 | 4 | 0 | 8 |
|  | Total | 381 | 141 | 12 | 534 |

^1^The minor allele (A) is the proxy for DR3
